# Supplementary material for: Experiences Shared by the (Future) Public Health Workforce during the COVD-19 Pandemic in Germany: Results of a Survey on Workload, Work Content, and Related Challenges among Students and Young Professionals
Source: Int J Environ Res Public Health. 2022 Sep 11;19(18):11444. doi: 10.3390/ijerph191811444 (PMC9517565; doi:10.3390/ijerph191811444)
Supplement: Supplementary file 1 [file ijerph-19-11444-s001.zip › ijerph-1868702-supplementary.pdf]

Supplementary materials

Table S1. Experiences of students enrolled for more than 3 semesters, stratified for gender, age, number of semesters absolved, and graduation status (n=76).

	**Gender**			**Age in years**				**Semesters absolved**			**Graduation during COVID-19 pandemic**		
	**Male**	**Female**	**SMD**	**≤27**	**28–<38**	**≥38**	**SMD**	**4-10**	**≥11**	**SMD**	**yes**	**no**	**SMD**
**n**	16	57		43	29	4		48	28		21	53	
**Money**			0.493				0.913			0.403			0.504
very burdened	0 (0.0)	3 (5.5)		1 (2.4)	2 (7.1)	0 (0.0)		1 (2.2)	2 (7.4)		0 (0.0)	3 (6.0)	
burdened	1 (5.6)	8 (14.5)		6 (14.6)	3 (10.7)	0 (0.0)		5 (10.9)	4 (14.8)		3 (14.3)	6 (12.0)	
less burdened	4 (22.2)	13 (23.6)		12 (29.3)	5 (17.9)	0 (0.0)		13 (28.3)	4 (14.8)		7 (33.3)	9 (18.0)	
not burdened	13 (72.2)	31 (56.4)		22 (53.7)	18 (64.3)	4 (100.0)		27 (58.7)	17 (63.0)		11 (52.4)	32 (64.0)	
NA=3													
**Lecturer**			0.511				1.045			0.182			0.436
very burdened	5 (27.8)	15 (26.3)		14 (32.6)	6 (21.4)	0 (0.0)		12 (25.0)	8 (29.6)		5 (23.8)	15 (28.8)	
burdened	7 (38.9)	25 (43.9)		16 (37.2)	13 (46.4)	3 (75.0)		22 (45.8)	10 (37.0)		7 (33.3)	25 (48.1)	
less burdened	2 (11.1)	13 (22.8)		8 (18.6)	7 (25.0)	0 (0.0)		9 (18.8)	6 (22.2)		6 (28.6)	8 (15.4)	
not burdened	4 (22.2)	4 (7.0)		5 (11.6)	2 (7.1)	1 (25.0)		5 (10.4)	3 (11.1)		3 (14.3)	4 (7.7)	
NA=1													
**Co-student**			0.447				1.071			0.043			0.316
very burdened	10 (55.6)	34 (59.6)		32 (74.4)	11 (39.3)	1 (25.0)		28 (58.3)	16 (59.3)		12 (57.1)	32 (61.5)	
burdened	4 (22.2)	18 (31.6)		7 (16.3)	14 (50.0)	1 (25.0)		14 (29.2)	8 (29.6)		7 (33.3)	14 (26.9)	
less burdened	2 (11.1)	4 (7.0)		4 (9.3)	1 (3.6)	1 (25.0)		4 (8.3)	2 (7.4)		2 (9.5)	4 (7.7)	
not burdened	2 (11.1)	1 (1.8)		0 (0.0)	2 (7.1)	1 (25.0)		2 (4.2)	1 (3.7)		0 (0.0)	2 (3.8)	
NA=1													
**Material**			0.374				0.960			0.504			0.339
very burdened	3 (16.7)	3 (5.3)		4 (9.3)	2 (7.1)	0 (0.0)		4 (8.3)	2 (7.4)		1 (4.8)	5 (9.6)	
burdened	5 (27.8)	17 (29.8)		12 (27.9)	10 (35.7)	0 (0.0)		14 (29.2)	8 (29.6)		7 (33.3)	14 (26.9)	
less burdened	5 (27.8)	18 (31.6)		15 (34.9)	7 (25.0)	1 (25.0)		18 (37.5)	5 (18.5)		5 (23.8)	18 (34.6)	
not burdened	5 (27.8)	19 (33.3)		12 (27.9)	9 (32.1)	3 (75.0)		12 (25.0)	12 (44.4)		8 (38.1)	15 (28.8)	
NA=1													
**Quiet place**			0.109				1.366			0.252			0.306
very burdened	7 (36.8)	21 (36.8)		18 (41.9)	10 (34.5)	0 (0.0)		16 (33.3)	12 (42.9)		9 (42.9)	19 (35.8)	
burdened	4 (21.1)	14 (24.6)		11 (25.6)	7 (24.1)	0 (0.0)		11 (22.9)	7 (25.0)		3 (14.3)	14 (26.4)	
less burdened	4 (21.1)	12 (21.1)		9 (20.9)	5 (17.2)	2 (50.0)		11 (22.9)	5 (17.9)		5 (23.8)	11 (20.8)	
not burdened	4 (21.1)	10 (17.5)		5 (11.6)	7 (24.1)	2 (50.0)		10 (20.8)	4 (14.3)		4 (19.0)	9 (17.0)	
NA=0													
**Something else**			0.713				NaN			1.088			1.014
very burdened	2 (66.7)	5 (50.0)		5 (62.5)	2 (40.0)	0 (NaN)		3 (37.5)	4 (80.0)		2 (40.0)	5 (62.5)	
burdened	1 (33.3)	3 (30.0)		2 (25.0)	2 (40.0)	0 (NaN)		3 (37.5)	1 (20.0)		2 (40.0)	2 (25.0)	
less burdened	0 (0.0)	1 (10.0)		1 (12.5)	0 (0.0)	0 (NaN)		1 (12.5)	0 (0.0)		0 (0.0)	1 (12.5)	
not burdened	0 (0.0)	1 (10.0)		0 (0.0)	1 (20.0)	0 (NaN)		1 (12.5)	0 (0.0)		1 (20.0)	0 (0.0)	
NA=63													

Legend: "During the COVID-19 pandemic, I experienced…"

Money: "…financing my student status as…"; Lecturer: "…lack of exchange with lecturers as…"; Co-student: "…lack of exchange with fellow students as…"; Material: "…lack of access to learning materials as…"; Quiet place: "…lack of access to undisturbed/quiet workplaces as…"; Something else: "…something else I would like mention as…".

NA: not applicable; SMD: standard mean difference
